# Supplementary material for: Partner choice, confounding and trait convergence all contribute to phenotypic partner similarity
Source: Nat Hum Behav. 2023 Mar 16;7(5):776–89. doi: 10.1038/s41562-022-01500-w (PMC10202815; doi:10.1038/s41562-022-01500-w)
Supplement: Supplementary file 1 — Supplementary Figs. 1–3. [file 41562_2022_1500_MOESM1_ESM.pdf]

nature human behaviour

Article https://doi.org/10.1038/s41562-022-01500-w

Partner choice, confounding and trait convergence all contribute to phenotypic partner similarity

In the format provided by the authors and unedited

Supplementary Figures

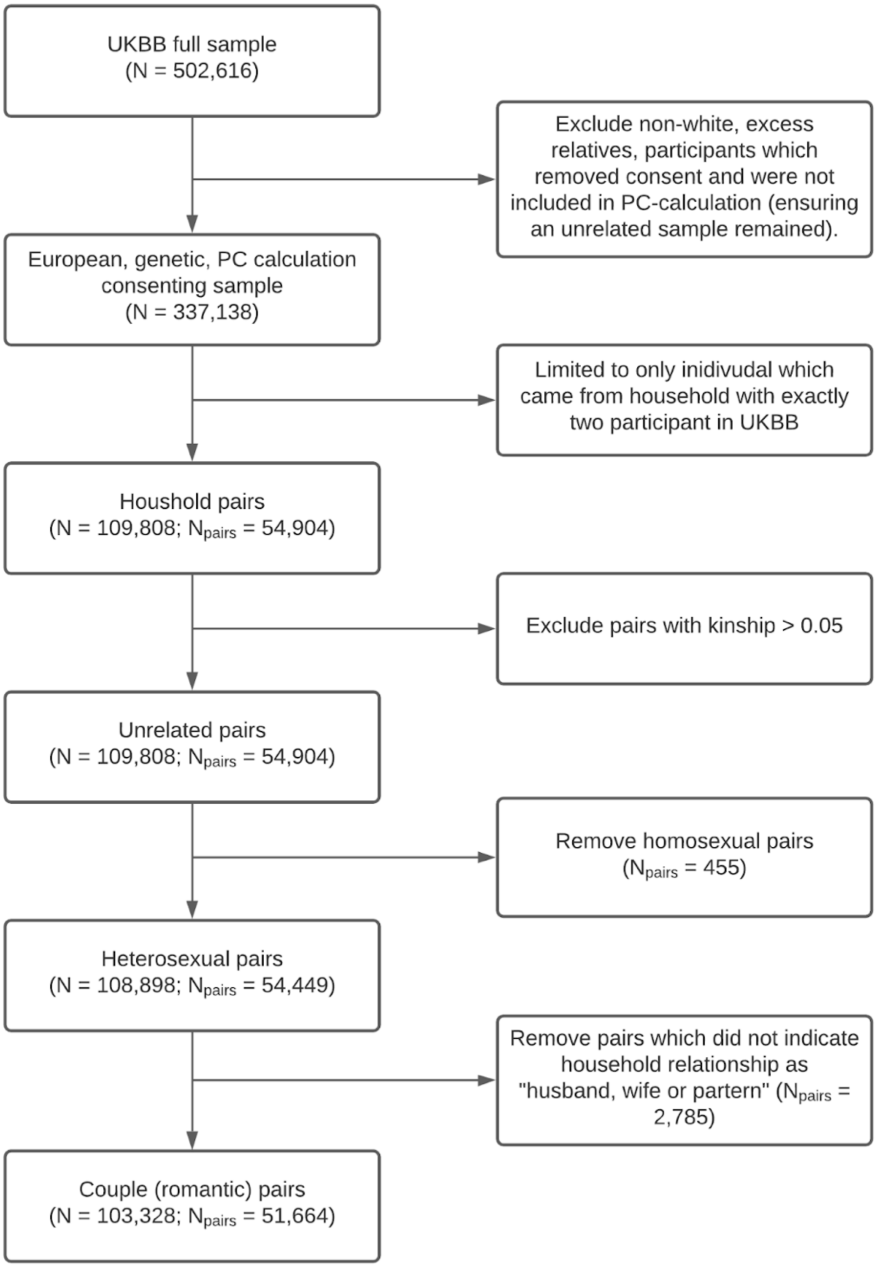

Supplementary Figure 1: Couple selection summary. *Flow chart shows a summary of the couple determination and selection in the UKBB. "Excess relatives" refers to an excessive number of relatives in the data set.*

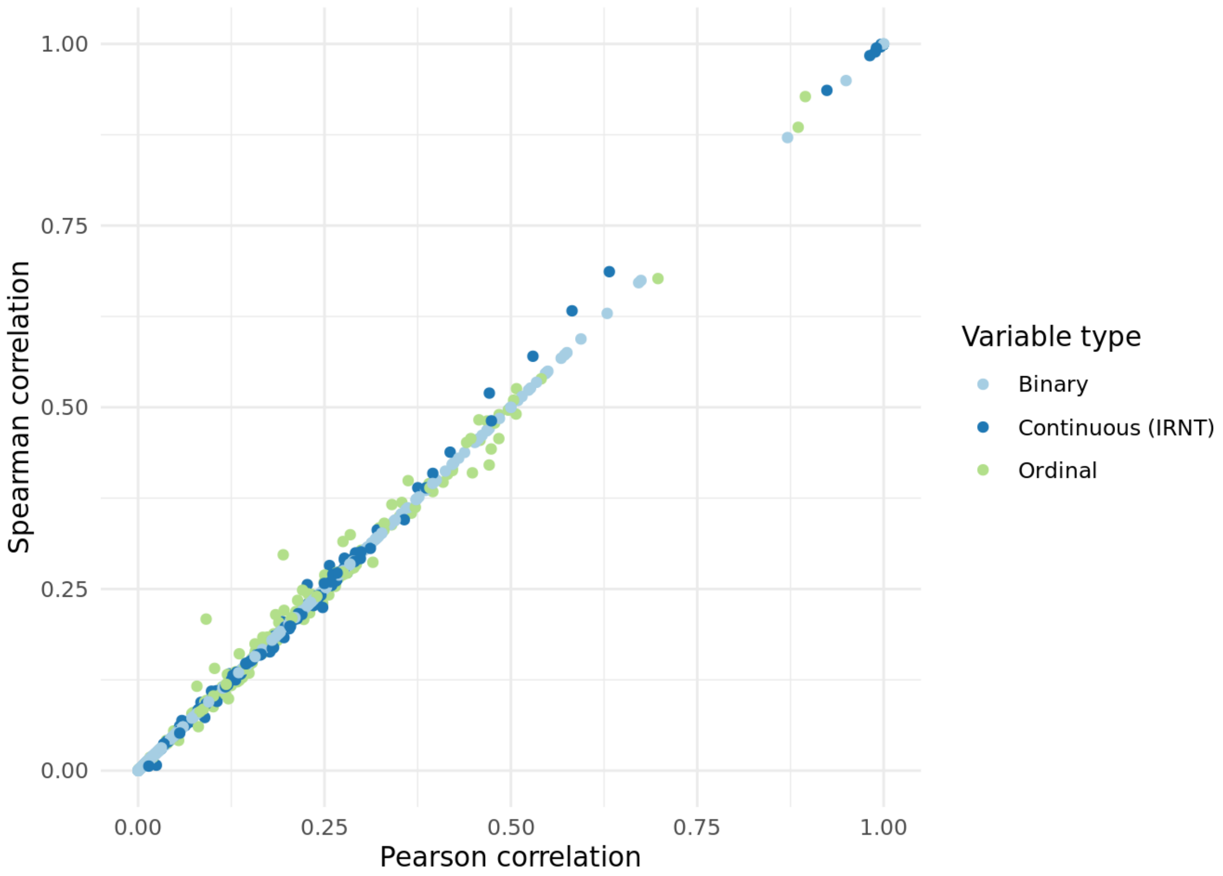

Supplementary Figure 2: Spearman versus Pearson correlation in the UKBB. *Scatter plot shows Spearman correlation versus Pearson correlation for each trait considered (n = 1278), color coded according to variable type. A reasonably good agreement can be observed between the two correlation estimates regardless of variable type.*

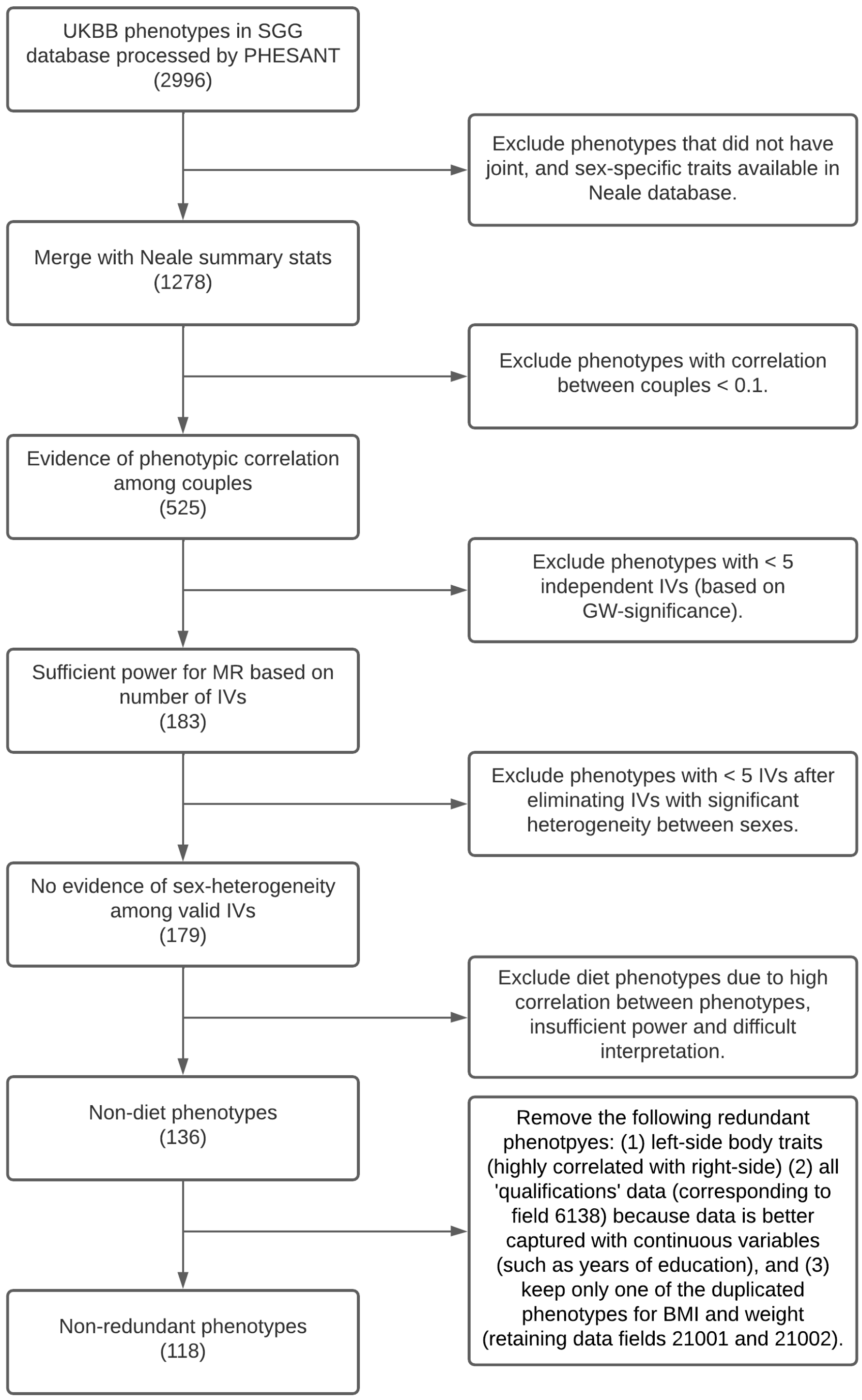

Supplementary Figure 3: Phenotype selection summary. *Flow chart shows a summary of the phenotype selection included in the pipeline, resulting in 118 phenotypes for analysis. SGG refers to the Statistical Genetics Group, data was used from the corresponding internal UKBB database under application number #16389.*
