# Supplementary material for: Partner choice, confounding and trait convergence all contribute to phenotypic partner similarity
Source: Nat Hum Behav. 2023 Mar 16;7(5):776–89. doi: 10.1038/s41562-022-01500-w (PMC10202815; doi:10.1038/s41562-022-01500-w)
Supplement: Supplementary file 2 — Reporting Summary [file 41562_2022_1500_MOESM2_ESM.pdf]

nature portfolio

Corresponding author(s): Zoltan Kutalik

Last updated by author(s): 17/11/2022

Reporting Summary

Nature Portfolio wishes to improve the reproducibility of the work that we publish. This form provides structure for consistency and transparency in reporting. For further information on Nature Portfolio policies, see our Editorial Policies and the Editorial Policy Checklist.

Statistics

For all statistical analyses, confirm that the following items are present in the figure legend, table legend, main text, or Methods section.

n/a	Confirmed	
☐	☒	The exact sample size (n) for each experimental group/condition, given as a discrete number and unit of measurement
☐	☒	A statement on whether measurements were taken from distinct samples or whether the same sample was measured repeatedly
☐	☒	The statistical test(s) used AND whether they are one- or two-sided<br>*Only common tests should be described solely by name; describe more complex techniques in the Methods section.*
☐	☒	A description of all covariates tested
☐	☒	A description of any assumptions or corrections, such as tests of normality and adjustment for multiple comparisons
☐	☒	A full description of the statistical parameters including central tendency (e.g. means) or other basic estimates (e.g. regression coefficient) AND variation (e.g. standard deviation) or associated estimates of uncertainty (e.g. confidence intervals)
☐	☒	For null hypothesis testing, the test statistic (e.g. F, t, r) with confidence intervals, effect sizes, degrees of freedom and P value noted<br>*Give P values as exact values whenever suitable.*
☒	☐	For Bayesian analysis, information on the choice of priors and Markov chain Monte Carlo settings
☒	☐	For hierarchical and complex designs, identification of the appropriate level for tests and full reporting of outcomes
☐	☒	Estimates of effect sizes (e.g. Cohen's d, Pearson's r), indicating how they were calculated

Our web collection on statistics for biologists contains articles on many of the points above.

Software and code

Policy information about availability of computer code

Data collection	No software was used for data collection.
Data analysis	Data analysis was performed using code written in the freely available R programming language (R version 3.6.3 (2020-02-29)) and is available at https://github.com/jennysjaarda/proxyMR. The cutr package was downloaded from https://github.com/moodymudskipper/cutr.

For manuscripts utilizing custom algorithms or software that are central to the research but not yet described in published literature, software must be made available to editors and reviewers. We strongly encourage code deposition in a community repository (e.g. GitHub). See the Nature Portfolio guidelines for submitting code & software for further information.

Data

Policy information about availability of data

All manuscripts must include a data availability statement. This statement should provide the following information, where applicable:

- Accession codes, unique identifiers, or web links for publicly available datasets
- A description of any restrictions on data availability
- For clinical datasets or third party data, please ensure that the statement adheres to our policy

The UK Biobank data is available through the standard UK Biobank application procedure (https://www.ukbiobank.ac.uk/enable-your-research/apply-for-access). The household information can be separately requested from the UK Biobank access team, as it is not part of the variables listed on the showcase (https://biobank.ndph.ox.ac.uk/showcase/index.cgi). The 1000 Genomes European genetic data can be downloaded from http://ftp.1000genomes.ebi.ac.uk/vol1/ftp/. Simulated data can be produced by executing the provided code.

Field-specific reporting

Please select the one below that is the best fit for your research. If you are not sure, read the appropriate sections before making your selection.

☒ Life sciences ☐ Behavioural & social sciences ☐ Ecological, evolutionary & environmental sciences

For a reference copy of the document with all sections, see nature.com/documents/nr-reporting-summary-flat.pdf

Life sciences study design

All studies must disclose on these points even when the disclosure is negative.

Sample size	All available (N=51,664) couples in the UK Biobank after exclusion described in Supplementary Figure 1.
Data exclusions	Non-British individuals, or those with missing data were excluded from the analysis. Further exclusion criteria are fully described in Supplementary Figure 1.
Replication	No sufficiently large other data set was available for replication. All analysis results can be reproduced based on the provided code.
Randomization	We had no experimental groups.
Blinding	There was no group allocation.

Reporting for specific materials, systems and methods

We require information from authors about some types of materials, experimental systems and methods used in many studies. Here, indicate whether each material, system or method listed is relevant to your study. If you are not sure if a list item applies to your research, read the appropriate section before selecting a response.

Materials & experimental systems

n/a	Involved in the study	
☒	☐	Antibodies
☒	☐	Eukaryotic cell lines
☒	☐	Palaeontology and archaeology
☒	☐	Animals and other organisms
☐	☒	Human research participants
☒	☐	Clinical data
☒	☐	Dual use research of concern

Methods

n/a	Involved in the study	
☒	☐	ChIP-seq
☒	☐	Flow cytometry
☒	☐	MRI-based neuroimaging

Human research participants

Policy information about studies involving human research participants

Population characteristics	Described in the UK Biobank protocol: https://www.ukbiobank.ac.uk/wp-content/uploads/2011/11/UK-Biobank-Protocol.pdf
Recruitment	See above.
Ethics oversight	This study used the UK Biobank (UKBB) cohort, a prospective population-based study with over 500,000 adult participants. UK Biobank has approval from the North West Multi-centre Research Ethics Committee (MREC) as a Research Tissue Bank (RTB) approval. This approval means that researchers do not require separate ethical clearance and can operate under the RTB approval. UK Biobank also possesses a Human Tissue Authority (HTA) licence, so a separate HTA licence is not required by researchers who receive samples from the resource.

Note that full information on the approval of the study protocol must also be provided in the manuscript.
